# Effects of botulinum toxin A on endometriosis-associated pain and its related mechanism

**DOI:** 10.3892/mmr.2020.11674

**Published:** 2020-11-09

**Authors:** Fubo Tian, Wuzhong Cheng, Jianying Hu, Shaoqiang Huang, Shen Sun

Mol Med Rep 22: 4351-4359, 2020; DOI: 10.3892/mmr.2020.11501

Following the publication of this paper, an interested reader drew to the attention of the Editorial Office that there were potentially concerns regarding the manner in which the botulinum toxin animal studies had been performed, and also in terms of the novelty of the study, wherein the authors had claimed that their study was the first to have explored the use of botulinum toxin for endometriosis-related pain.

Having asked the authors to comment on these points, they have conceded that the animal experiments, which were performed 5 years previously, may have been flawed from the perspective of the methodology, although the group are no longer able to contact the person who performed the experiments. Furthermore, the authors have subsequently re-reviewed the field of botulinum toxin usage in endometriosis, and concede that their study has made only an incremental advance in knowledge in this area. Therefore, on the grounds that this study may have contained procedural errors in the animal experiments which the authors were unable to verify on account of having lost contact with the person who performed them, and in view of the misinformation regarding the novelty of the study, the authors have requested that the paper be retracted from the publication. The Editor of *Molecular Medicine Reports* has agreed that the paper should be retracted; moreover, the authors apologize to the readership for any inconvenience caused.

